# Ornithine transcarbamylase deficiency combined with type 1 diabetes mellitus - a challenge in clinical and dietary management

**DOI:** 10.1186/2251-6581-12-37

**Published:** 2013-07-05

**Authors:** Sarah C Grünert, Pablo Villavicencio-Lorini, Bendicht Wermuth, Willy Lehnert, Jörn Oliver Sass, K Otfried Schwab

**Affiliations:** 1 Center of Pediatrics and Adolescent Medicine, University Hospital Freiburg, Freiburg, Germany; 2 Institute of Clinical Chemistry Laboratory, University of Bern, Bern, Switzerland; 3 Division of Clinical Chemistry and Biochemistry, University Children’s Hospital Zürich, Zürich, Switzerland

**Keywords:** Ornithine transcarbamylase deficiency, Ornithine carbamoyltransferase deficiency, OTC deficiency, Urea cycle disorder, Insulin-dependent diabetes mellitus, Hyperammonemia, Insulin

## Abstract

Ornithine transcarbamylase (OTC) deficiency is the most common urea cycle defect. The clinical presentation in female manifesting carriers varies both in onset and severity. We report on a female with insulin dependent diabetes mellitus and recurrent episodes of hyperammonemia. Since OTC activity measured in a liver biopsy sample was within normal limits, OTC deficiency was initially excluded from the differential diagnoses of hyperammonemia. Due to moderately elevated homocitrulline excretion, hyperornithinemia-hyperammonemia-homocitrullinuria-syndrome was suggested, but further assays in fibroblasts showed normal ornithine utilization. Later, when mutation analysis of the *OTC* gene became available, a known pathogenic missense mutation (c.533C>T) in exon 5 leading to an exchange of threonine-178 by methionine (p.Thr178Met) was detected. Skewed X-inactivation was demonstrated in leukocyte DNA. In the further clinical course the girl developed marked obesity. By initiating physical activities twice a week, therapeutic control of both diabetes and OTC deficiency improved, but obesity persisted. In conclusion, our case confirms that normal hepatic OTC enzyme activity measured in a single liver biopsy sample does not exclude a clinical relevant mosaic of OTC deficiency because of skewed X-inactivation. Mutation analysis of the *OTC* gene in whole blood may be a simple way to establish the diagnosis of OTC deficiency. The joint occurrence of OTC deficiency and diabetes in a patient has not been reported before.

## Background

Ammonia which is predominantly formed by degradation of amino acids is bound as urea by action of the urea cycle. Urea cycle defects (UCDs) are a group of inborn errors of metabolism comprising 6 enzymatic defects (five core enzymes and one activating enzyme) as well as a mitochondrial ornithine/citrulline antiporter defect. Among UCDs, ornithine transcarbamylase (ornithin carbamoyltransferase, OTC, E.C. 2.1.3.3) deficiency (OMIM #311250) is the most prevalent with an incidence of 1:14.000 newborns [1-4]. OTC, a mitochondrial matrix enzyme, catalyzes the biosynthesis of citrulline from ornithine and carbamoyl phosphate. The corresponding gene is located on Xp11.4. While hemizygote males usually present with severe neonatal hyperammonemic coma which often proves to be fatal [2,5], the clinical picture in female carriers of this X-linked disease is extremely variable, depending on the degree of inactivation of the mutated X chromosome.

Survival is better among those who have late onset of symptoms like some female carriers or males with only slightly reduced OTC activity [6-8]. While neonatal onset cases are usually diagnosed reliably using biochemical parameters (like excretion of large amounts of orotic acid and uracil and a typical amino acid pattern with elevated glutamine and very low or not detectable citrulline levels) diagnosis may be more complicated in late onset cases [9]. The mainstay of dietary treatment is protein restriction with supplementation of essential amino acids to minimize nitrogen load [9]. An adequate intake of calories is necessary to prevent protein catabolism [10]. In order to deplete the hepatic nitrogen pool, oral sodium benzoate and/or sodium phenylbutyrate can be applied to eliminate ammonia without stressing the urea cycle. The only curative treatment of OTC deficiency available is liver transplantation [9,11-15]. In this article we report the joint occurrence of OTC deficiency and type 1 diabetes and describe the diagnostic pitfalls and challenges of dietary therapy.

## Case presentations

We report a female, the first child of non-consanguineous German parents, who was born without complications following an uneventful pregnancy. At 7 weeks of age she had an episode of vomiting when formula replaced breast feeding. She was further breastfed until 6 months. She then received an adapted cow’s milk formula. At the age of 9 months loss of psychomotor abilities was observed together with fatigue, repeated vomiting and diarrhoea. At the age of 11 months the child was hospitalized. Her height was 75 cm (50th percentile) and her weight 8.5 kg (25th percentile). Liver and spleen were slightly enlarged. Reduced spontaneous movements and truncal hypotonia were noticed. The child could neither sit nor crawl nor pull herself up to stand. CT of the brain showed moderate cortical atrophy. Laboratory investigations revealed elevated transaminases and hyperammonemia (Table 1). Additional diagnostic parameters including amino acid concentrations measured in plasma and urine and orotic acid excretion (Table 2) suggested OTC deficiency. Protein restriction (0.93 g/kg/day of natural protein plus 0.5 g/kg/day of synthetic protein/essential amino acids) was instituted and transaminases normalized progressively within 2 weeks and ammonia after one month (Table 1). In order to verify OTC deficiency a liver needle biopsy was performed. Liver histology revealed pericentral fine-meshed fibrosis with faint portal filament augmentation without signs of inflammation. Low-grade intracellular accumulation of fat droplets of variable sizes was noticed. Unexpectedly, OTC activity in hepatic tissue was normal (261 nmol/min/mg liver protein, normal values >160 nmol/min/mg liver protein). Therefore, by exclusion, hyperornithinemia-hyperammonemia-homocitrullinuria syndrome (HHH syndrome, OMIM #238970) was considered, especially, because homocitrulline was intermittently detectable in urine. However, ornithine concentrations were always within the normal range, even during a 7-day trial of ornithine supplementation for diagnostic purposes. Substitution with citrulline (200–400 mg/kg/d according to arginine level) was introduced. Waste nitrogen excretion by alternate pathways was enhanced by sodium benzoate and lactulose was given to limit reabsorption of ammonia in the gut. Under this regimen, the girl remained clinically stable during the following years except for several metabolic decompensations with only mild hyperammonemia. The physical and psychomotor development was normal.

**Table 1:** Decline of ALAT, ASAT and ammonia after starting of a protein-restricted diet at the age of 11 months

**Day**	**Ammonia (μmol/l)**	ALAT (U/l)^*^	ASAT (U/l)^*^
	**normal range 15–55 μmol/l**	**normal range < 22 U/l**	**normal range < 15 U/l**
**1**	365	516	444
**2**	360	1215	1010
**8**	**start of protein restriction (0.93 g/kg/d)**		
**15**	52	122	26
**34**	38	25	22
**44**	45	11	16

*measured at 25°C.

**Table 2:** Diagnostic parameters in plasma and urine before treatment at the age of 11 months

**Parameter**	**Patient results**	**Reference range**
Ornithine in plasma	30 μmol/l	39-61 μmol/l
Glutamine in plasma	2100 μmol/l	60-470 μmol/l
Arginine in plasma	70 μmol/l	53-71 μmol/l
Citrulline in plasma	not detected	no reference range given
Lysine in plasma	500 μmol/l	107-163 μmol/l
Homocitrulline in urine	17 mmol/mol creatinine	not detectable
Orotic acid in urine	> 8280 mmol/mol creatinine	1.3-8.5 mmol/mol creatinine

At the age of 4 years, the girl developed insulin-dependent diabetes (islet-cell antibodies positive, but insulin antibodies negative). The treatment had now to include carbohydrate control and insulin injections following a 2 insulin injection regime. The further clinical course was characterized by intermittent periods of both hyperammonemia and ketoacidosis, which were readily controlled by sodium benzoate (up to 350 mg/kg/d), arginine-HCl (2 mmol/kg/d) and glucose-insulin infusions as well as oral phenylbutyrate (100–300 mg/kg/d) during several hospital stays. Later on, diabetes management was switched to intensive insulin therapy (4 insulin injection regime). Nevertheless, diabetes treatment was suboptimal and HbA_1c_ levels were intermittently elevated up to 111 mmol/mol Hb (normal 20–42 mmol/mol Hb).

**Figure 1:**
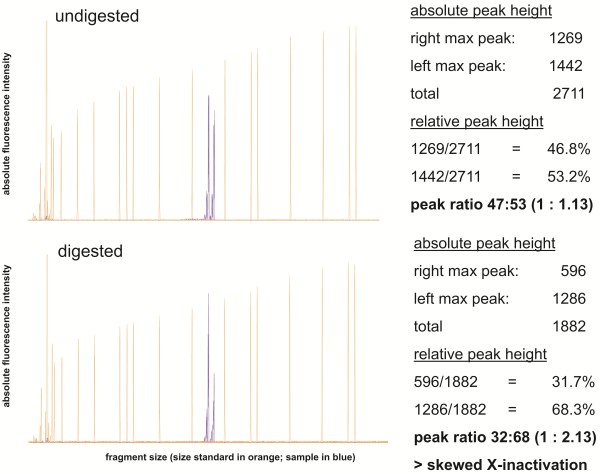
**X-inactivation analysis demonstrating skewed inactivation in the female patient, assessed from leukocyte DNA using the HUMARA method.** X-chromosomal alleles from an undigested control sample are shown in the upper panel. By HpaII digestion of a second sample, which leads to restriction of unmethylated DNA, an unequal methylation status and thereby skewed X-inactivation of the X-chromosomal alleles can be identified, as shown in the lower panel.

Since homocitrullinuria was inconsistent and always remained discrete, the suggested diagnosis was challenged. In order to prove or disprove the diagnosis of HHH syndrome ^14^C-ornithine incorporation in fibroblasts was measured (^14^C-ornithine/^3^H-leucine ratio 0.368; controls 0.392; 0.401; known HHH syndrome: ^14^C-ornithine/^3^H-leucine ratio 0.033). The normal result excluded a defect of the SLC25A15 ornithine transporter and thereby HHH syndrome. Because of marked increases of orotic acid excretion especially during metabolic decompensations, OTC deficiency was again suspected despite normal liver OTC activity. Mutation analysis of the *OTC* gene, which had not yet been available at the time of OTC enzyme analysis, confirmed carrier status of OTC deficiency with a known pathogenic heterozygous missense mutation in the *OTC* gene (c.533C>T, p.Thr178Met). Furthermore, a skewed X-inactivation with a ratio of (1:2.13) was noted in leukocyte DNA (Figure 1). This finding supports the suspicion that a clinical relevant hepatic mosaic of OTC deficiency might have been missed by a single liver biopsy. The changed diagnosis did not alter the dietary therapy and medication. The patient’s mother was also investigated genetically, but no *OTC* mutation could be detected.

As a complication of her diet, the girl developed progressive obesity. At the age of 15 years, she reached a BMI of 32.3 kg/m^2^. At that time she started a sports program with other diabetic adolescents and performed physical activities twice a week. Subsequently, her diabetes control improved, HbA_1c_-levels decreased from 86 mmol/mol Hb to 60 mmol/mol Hb and serum fructosamine levels from 497 to 290 μmol/l (normal 208–269 μmol/l). Moreover, during that time no hospitalizations because of hyperammonemia or ketoacidosis were necessary. However, during the following years, her compliance to therapy was not always satisfactory and HbA_1c_ values ranged from 62–89 mmol/mol.

The patient finished school and became a nurse. Her psychomotor development is normal. IQ-testing at the age of 26 years revealed an IQ of 94 (Wechsler Intelligenztest für Erwachsene). Now, at the age of 27 years, she lives an independent adult live and works as a nurse in an intensive care unit.

## Methods

The ornithine transporter assay was performed as described before by Shih et al. [16]. Liver enzyme activity of OTC was determined according to Raijman [8].

Genomic DNA was isolated from peripheral blood leukocytes using proteinase K/sodium dodecyl sulfate treatment and phenol/dichloromethane extraction [17].

Mutation analysis of the *OTC* gene was performed by bidirectional sequencing of all 10 exons including adjacent intronic sequences. Exons 7 and 8 were amplified as a single PCR product including intron 7 (80 bp).

X-inactivation analysis was performed by a PCR methylation assay for the human androgen receptor (*HUMARA*) gene locus comparing HpaII digested versus undigested samples, as described previously [18]. PCR products of X-chromosomal alleles were analyzed on a ABI3130xl capillary sequencer (Applied Biosystems) and visualized by Peak Scanner Software v1.0 (Applied Biosystems). For each sample, absolute values for the major peak heights of the two alleles were determined (ignoring any stutter peaks) and converted into relative peak heights, based on the sum of both allele peaks as 100%. Peak ratios were calculated by dividing the signal of the allele with the longer fragment size by the signal of the allele with the shorter fragment size.

## Discussion

OTC deficient males are often severely affected [5] whereas female carriers present with a wide phenotypic variability [19,20]. Some may be entirely asymptomatic, while others have severe hyperammonemia leading to brain damage or death [9,21]. This clinical variability is explained by X-inactivation in the liver. In females, X-inactivation is a random process inactivating either the maternal or the paternal allele. If X-inactivation results in predominant expression of the mutated allele, female carriers may be affected due to decreased OTC enzyme activity. In our patient a skewed X-inactivation pattern was found in leukocytes (see Figure 1). It has been shown by Yorifuji et al. that the X-inactivation pattern may differ significantly even within the same hepatic tissue but correlates well with the OTC activity in all liver samples [21]. Thus, it was concluded that a single liver biopsy is not adequate for the assessment of residual enzyme activity in females. This is underlined by our case where normal OTC activity was detected in a single liver biopsy despite symptomatic OTC deficiency. Therefore, according to the recently published guidelines for the diagnosis of urea cycle disorders [9] mutation analysis of the OTC gene is the diagnostic gold standard and permits to identify carrier females. However, in about 20% of patients the mutation is not identified with standard techniques [22]. The c.533C>T mutation found in our patient was first described in a male suffering from severe neonatal onset OTC deficiency [23]. In the majority of female patients and in some male patients the mutation appears *de novo* and the mother is not a disease carrier [9], as was the case in our patient.

To our knowledge, this is the first report on a symptomatic female with OTC deficiency with additional insulin-dependent diabetes.

In view of the two underlying diseases, a vicious cycle aggravating metabolic decompensations in our patient can be hypothesized: In case of insulin deficiency, glucose cannot enter the cells properly, thus leading to energy deficiency and catabolism. Apart from endogeneous protein catabolism resulting in hyperammonemia, increased hepatic glucose production and decreased peripheral glucose utilization may lead to hyperglycemia. The increased blood glucose levels, in turn, might result in glucosuria and loss of calories further enhancing catabolism and thereby aggravating hyperammonemia. Several hospital admissions were necessary for induction of anabolism by intravenous glucose-insulin and fat infusions, but also for intensified detoxification of ammonia by sodium benzoate and phenylbutyrate supplementation.

There is a large body of evidence that, on average, diabetic patients have a higher body weight and body mass index compared to healthy controls [24] and therefore should control their calorie intake. On the other hand, for patients with urea cycle disorders a sufficient calorie intake is required to prevent catabolism. The diet of our patient was complicated by the restriction of both carbohydrates and protein due to her diabetes and OTC deficiency, respectively. Subsequently, the resulting increased fat intake may have contributed to obesity. Regular physical activity did not only improve the patient’s diabetes control, reflected by a marked decrease in HbA_1c_ levels and stabilization of blood glucose levels, but also her metabolic control. Moderate physical exercise may induce protein anabolism and simultaneously lower glucose levels.

## Conclusion

In conclusion, our case report illustrates that liver OTC activity assays are not reliable in the diagnosis of OTC deficiency in females due to the mosaicism caused by X-inactivation. Mutation analysis of the *OTC* gene is a safer and less invasive approach to confirm the diagnosis. Since OTC deficiency and diabetes have different dietary implications the treatment is challenging.

## Consent

Written informed consent was obtained from the patient for publication of this Case report and any accompanying images. A copy of the written consent is available for review by the Editor-in-Chief of this journal.

## Abbreviations

ALAT: alanine aminotransferase; ASAT: *aspartate aminotransferase*; HbA1c: Glycosylated hemoglobin A; HHH syndrome: Hyperornithinemia-hyperammonemia-homocitrullinuria syndrome; OTC: Ornithine transcarbamylase.

## Competing interests

The authors declare that they have no competing interests.

## Authors’ contributions

SCG was involved in the treatment of the patient and drafted the manuscript with KOS. PVL was responsible for the X-inactivation studies and critically revised the manuscript. BW was responsible for the enzyme studies and critically revised the manuscript. WL was involved in the diagnostic work-up of the patient, responsible for most of the biochemical analyses and critically revised the manuscript. JOS was also involved in the biochemical investigations and critically revised the manuscript. KOS was involved in the diagnostic work-up and the long-term treatment of the patient. He drafted the manuscript together with SCG. All authors read and approved the final manuscript.
